# Exosomes derived from Umbilical cord mesenchymal stem cell promote hair regrowth in C57BL6 mice through upregulation of the RAS/ERK signaling pathway

**DOI:** 10.1515/jtim-2024-0012

**Published:** 2024-11-06

**Authors:** Yongcui Mao, Pinyan Liu, Jiayun Wei, Ye Xie, Qiuxia Zheng, Xuekai Hu, Jia Yao, Wenbo Meng

**Affiliations:** First Hospital of Lanzhou University, Lanzhou, Gansu Province, China; School of Pharmacy, Lanzhou University, Lanzhou, Gansu Province, China; Department of General Surgery, the First Hospital of Lanzhou University, Lanzhou, Gansu Province, China; Key Laboratory of Biotherapy and Regenerative Medicine of Gansu Province, Lanzhou, Gansu Province, China

**Keywords:** androgenetic alopecia, dihydrotestosterone, stem cell-derived exosomes, mitogen-activated protein kinase pathway

## Abstract

**Background and Objectives:**

Androgenetic alopecia is one of the common types of hair loss and has become a medical and social problem due to its increasingly young onset. Existing therapies, although effective, have serious side effects and therefore better treatments need to be sought. The aim of this study was to evaluate the efficacy of umbilical cord mesenchymal stem cell-derived exosomes in the treatment of androgenetic alopecia and to investigate the mechanism of exosome regulation of hair growth.

**Methods:**

First, we randomly divided 20 C57BL/6J mice into blank group, model group, positive control group and exosomal hydrogel group, and mice were treated with hair removal on the back. The mice were injected intraperitoneally with dihydrotestosterone solution except for the blank group. At the end of the experiment, new hairs were collected and the differences in length, diameter and number of hair follicles were compared among the groups; the histopathological changes of hair follicles were observed by HE staining; the expression of androgen receptor mRNA and protein in skin tissues were compared; and the skin tissues were analyzed by real-time PCR, western blotting, immunofluorescence staining and transcriptome sequencing. Finally, the results of transcriptome sequencing experiments were verified by real-time PCR, western blotting and other techniques for the corresponding genes and proteins.

**Results:**

Compared with the blank group, mice in the model group had shorter hair length and reduced hair diameter, and pathological observation showed that the total number of hair follicles was significantly reduced and the hair follicles were miniaturized; compared with the model group, mice in the positive control and exosome groups had longer hair length, larger hair diameter and more hair follicles; the androgen receptor mRNA content and protein expression in the skin tissue of mice in the model group were significantly higher than those in the blank group, and the protein expression in the exosome gel group was lower than that in the model group. Similarly, compared with the model group, the expression of stemness-related proteins K15 and CD200 in the skin tissues of mice in the exosome group increased, and the expression of PCNA, a protein related to cell proliferation, increased. The KEGG data showed that the differential genes were mainly enriched in the RAS/ERK pathway.

**Conclusions:**

In this study, we demonstrated the therapeutic effect of umbilical cord MSC-derived exosomes on androgenetic alopecia and verified that exosomes regulate hair follicle stem cell stemness through the RAS/ERK pathway to promote hair proliferation and thus hair growth in mice with androgenetic alopecia, providing a potential therapeutic strategy for androgenetic alopecia.

## Introduction

Androgenetic alopecia (AGA), also known as seborrheic alopecia, is a common condition characterized by non-scarring progressive follicular shrinkage that occurs in a distribution pattern in susceptible men and women. AGA affects approximately 50% of menopausal women, 35% of women of childbearing age, and 70% of adult men.^[1]^ Although AGA is not life-threatening, it severely affects the quality of life and self-esteem of patients and currently has very limited approved treatment options. The objective of AGA treatment is to halt the progression and prevent further miniaturization. However, patients’ expectations of treatment outcomes may exceed the reality, and treatment may not always result in improvement or regrowth.^[2]^

Current treatments for AGA encompass a range of options, including topical and oral medications, plasma platelet-rich (PPR) injections, low-laser therapy, microneedling, and hair transplant surgery. Among the drugs approved by the Food and Drug Administration (FDA) for hair loss, minoxidil and finasteride are commonly used; however, their success rates are only 35% and 48%, respectively.^[3]^ Finasteride, a 5α-reductase inhibitor, exhibits positive effects in treating patients with AGA. Nevertheless, this medication is associated with the potential to induce male sexual dysfunction attributed to its effects on testosterone and dihydrotestosterone (DHT).^[4]^ In contrast, oral minoxidil has been found to be an effective and well-tolerated treatment alternative to finasteride for difficult treatment modalities.^[5]^ Recently, in August 2020, the FDA approved Clasto Progesterone Cream 1% for the first time in the United States as a topical treatment for acne vulgaris in patients 12 years of age or older. However, clinical studies of a different formulation of Clasto Progesterone for the treatment of AGA are currently underway in Germany and the United States.^[6]^

Previous studies have shown a moderate effect of PPR therapy on hair growth in patients with AGA; however, the lack of standardized PPR formulations and treatment protocols hinders the comparison of study results.^[7]^ Low-level laser therapy (LLLT) has been shown to improve hair regrowth, hair thickness, and patient satisfaction. Two commercially available LLLT devices, the Hair Max Laser Comb (Lexington Int. LLT, Boca Raton, FL, USA) and TOPHAT 655 (Apira Science Inc., Boca Raton), are FDA-approved due to their minimal risk.^[8]^ Microneedling, a minimally invasive method for treating AGA, involves rolling fine needles over the skin to pierce the stratum corneum and induce collagen formation, neovascularization, and growth factor production in the treated area.^[9]^ Although the number of studies investigating this therapy for hair loss is limited, microneedling has been successfully paired with other hair growth-promoting therapies, such as minoxidil, PPR, and topical steroids, and has been shown to stimulate hair follicle (HF) growth, suggesting that microneedling facilitates the penetration of this first-line drug as a mechanism to promote hair growth.^[10]^ Hair transplantation is considered a potentially advantageous treatment for AGA, and its efficacy hinges on the availability of sufficient hair in the patient’s donor area, which is essential to procure an adequate quantity of grafts capable of providing coverage for the affected region of alopecia. It has been reported that graft survival rates and the satisfaction level of patients who underwent this procedure are approximately 86% to 88%.^[11]^ Despite the efficacy of these treatments, each has its limitations, and novel strategies are required to treat hair loss.

Although AGA pathogenesis is still controversial, it is generally associated with DHT expression. The accumulation of DHT in androgen-sensitive HFs leads to a higher DHT expression in the scalp tissues of individuals with alopecia areata than in those without. [12] Type II 5-α reductase converts free testosterone into DHT, which shrinks the HFs, resulting in thinner and lighter hair, a shortened hair growth cycle, and eventually leading to atrophy and HFs loss.^[13]^ Recent studies have focused on hair follicle stem cells (HFSCs), which are located in the raised region of the outer root sheath of the HF and possess multilineage differentiation potential and superior proliferative capacity. Normal morphology and cyclic growth of HFSCs are critical for maintaining normal skin function, wound repair, and skin regeneration.^[14]^ The growth of HFs is propelled by circulating HFSCs, and stem cell activity is non-cell-autonomously regulated by the local microenvironment or ecological niche.^[15]^ To adapt to different physiological conditions and changing external environments, stem cell ecotopes have evolved multiple functions that allow stem cells to perceive these changes and communicate with remote cells/tissues to accommodate the needs of the organism.^[16]^

The field of regenerative medicine has seen a surge of interest in stem cell-based therapies, such as stem cell transplantation, stem cell-conditioned media, and stem cell-derived exosomes. While the efficacy of both autologous and allogeneic mesenchymal stem cells (MSCs) has been previously demonstrated,^[17]^ the use of conditioned media from MSCs in treating chronic wounds has shown similar or greater effectiveness and regenerative capacity than with using MSCs. Exosomes, a type of single-membrane vesicles with a similar topology as cells, are between 30–200 nm in diameter and contain a wide range of transmembrane proteins, lipid-anchored membrane proteins, peripheral-associated membrane proteins, and soluble proteins within their lumen.^[18]^ The MSC-derived exosome form of the secretome carries soluble factors and metabolites that play an essential role in the wound-healing process.^[19]^ Due to their ability to promote cell proliferation, exosomes are widely used in hair loss treatment research, and numerous preclinical studies have shown promising results.^[14]^ However, the underlying mechanism through which exosomes promote hair growth remains unclear. Therefore, we hypothesized that exosomes promote hair papilla cell proliferation by enhancing the stemness of HFSCs. To investigate this, we examined the mechanism by which umbilical cord MSCs exosomes promote hair growth in a mouse model of AGA.

## Materials and methods

### Extraction and identification of humanumbilical cord mesenchymal stem cells (humscs)

The collection and processing of discarded umbilical cords from primiparas were carried out at the First Hospital of Lanzhou University. Following informed consent from the patient, the umbilical cords were aseptically handled and transferred to umbilical cord preservation bottles and then immediately transported to the laboratory under a constant temperature of 4 °C. Disinfection of the umbilical cord was undertaken before a small segment was excised to remove the stasis, following which the remaining cord was sectioned into small pieces using sterile surgical scissors and inoculated into 75T culture flasks. The medium was supplemented with 15 mL of the prepared medium after 3 h of incubation, and the culture was maintained under standard incubation conditions at 5% CO_2_, saturated humidity, and 37 °C. Upon attainment of appropriate cell growth around the tissue block, Huc-MSCs were treated with primary antibodies (FITC-labeled anti-CD45 and anti-CD73, APC-labeled anti-CD105, and HLA-DR primary antibodies from BioLegend) and incubated for 30 min, washed, and resuspended in phosphate-buffered saline (PBS). The analysis was performed using a flow cytometer (BD, USA).

### Culture and supernatant collection of hUMSCs

The p3-p7 generation umbilical cord MSCs were cultured in a CO_2_ incubator at a standard temperature of 37 °C and a CO_2_ concentration of 5% using serum-containing medium (FBS: DMEM/F12 = 1: 9). Upon attaining 70%-80% cell confluency, the serum-containing medium was removed, and the cells were rinsed thrice with PBS and then maintained in serum-free medium (DMEM/F12) for 48-72 h. The resulting supernatant was collected and stored at –20 °C for further analysis.

### HUCMSCs-Exos extraction and identification

The collected supernatant was subjected to a series of centrifugation steps. Initially, the supernatant was centrifuged at 800 rpm for 5 min to remove live cells and some cell debris. The resulting supernatant was transferred to a new tube and centrifuged at 3000 × g for 15 min to remove most of the remaining cell debris. The supernatant was then transferred to an ultracentrifuge tube and subjected to an additional centrifugation step at 12000 × g for 10 min to further eliminate fine debris, impurities, and other particulate matter. The concentrated supernatant was then transferred to a 50-ml MWCO 100 kD ultrafiltration tube and centrifuged at a minimum rate of 2000 rpm for 20–30 min to concentrate the supernatant to 200-300 μL. Lastly, the collected supernatant was mixed with the reaction solution according to the protocol of ExoQuick-TC® Exosome Extraction Kit and gently pipetted with a pipette gun. The obtained exosomes were resuspended in 100-500 μL of PBS at 4 °C or -80 °C, and the protein quantity of hUCMSCs-Exos exosomes was measured using the bicinchoninic acid protein assay kit. The morphology, size, and marker expression (TSG101, CD63, and CD81) of hUCMSC-Exos were determined using conventional transmission electron microscopy in combination with nanoparticle tracking analysis (NTA) and western blotting.

### Preparation of exosome hydrogels

Temperature-sensitive hydrogels were prepared using the cold dissolution method. Poloxamer 407 (F20180001348, BASF), poloxamer 188 (F20180001349, BASF), and exosomes were accurately weighed and subsequently dissolved in an appropriate volume of pre-cooled ultrapure water. The mixture was thoroughly blended under ice water bath conditions and refrigerated at 4 °C overnight to ensure complete dissolution.

### Mice

The Ethics Committee of the First Hospital of Lanzhou University approved all the procedures involved in this study, as indicated by the approval document number LDYYLL2022-03. The study utilized SPF-grade male C57BL6 mice (6 weeks old and weighing 20 g) that were procured from the Lanzhou Veterinary Research Institute, Chinese Academy of Agricultural Sciences. Twenty mice were randomly allocated to four groups, namely, blank, model, positive control, and exosome hydrogel groups (exosome group), each containing five mice. After administering isoflurane gas anesthesia, a 1: 1 volume ratio mixture of heated and melted rosin (C832364-500g, Macklin) and paraffin (YA0020, Solarbio) was applied to the back of mice once the temperature dropped to approximately 50 °C. Upon cooling and solidification, the coat was removed, and the hair removal area was 2 cm × 3 cm. To establish an AGA model, DHT (A8960, Solarbio) was dissolved in corn oil (C7030, Solarbio) to form a 10 mg/mL solution and injected intraperitoneally (0.1 mL or 1 mg) daily for 17 days in all groups except the blank group. The positive control group was treated topically with 5% minoxidil (Zhejiang Wansheng Pharmaceutical Co., Ltd., China) twice daily on the hair loss area, while the exosome group was treated with 1 mL of gel twice daily on the same area. Seventeen days later, the mice were euthanized to assess the model and hair growth. The mice were anesthetized with isoflurane and maintained at body temperature during the photographic session. At the end of the experiment, the mice were sacrificed using the cervical dislocation method, and skin samples were collected and stored at-80 °C until further use.

### General observation indicators

The daily skin and hair growth of each group of mice were systematically observed after hair removal. Photographs were captured and recorded every 2 days to monitor the progress of hair growth in each group. To determine the hair diameter, one hundred hairs were collected randomly from each mouse and measured under a microscope (IX73, Olympus). Additionally, hair length was measured using Vernier calipers.

### Histological examination

The skin specimens were collected at the conclusion of the experiment and subsequently fixed in 4% paraformaldehyde (P1110, Solarbio) for 30 min. These specimens were then embedded in paraffin and sliced in longitudinal planes with HFs at a thickness of 45 μm. Hematoxylin staining solution (G1003, Servicebio) was utilized to stain the specimens for 3-5 min in tap water, after which they were immersed in differentiation solution, washed in tap water, and returned to blue in return blue solution before being rinsed in running water. The sections were then dehydrated in 85% and 95% gradient alcohol for 5 min each and stained with an eosin staining solution for 5 min. Subsequently, the samples were dehydrated in anhydrous ethanol and sealed with neutral gum (10004160; SCRC). Tissue morphology was observed under a microscope, with three samples selected from each group and 10 fields of view chosen from each group under low magnification. The number of HFs present in each field of view was counted, and the mean value was used to compare the differences in the number of HFs between the groups.

### Immunohistochemically and immunofuorescence staining

The collected mouse skin tissues were fixed in 4% solution of paraformaldehyde (P1110, Solarbio) for 30 min, embedded in paraffin, and extended HFs were sectioned longitudinally to a thickness of 45 μm. The tissue was subsequently dehydrated and sealed with citric acid antigen solution (G1202, Servicebio) to repair the antigen. Endogenous peroxidase was blocked using a 3% hydrogen peroxide solution. Subsequently, drops of 3% bovine serum albumin (BSA) (GC305010, Servicebio) were added to block non-specific binding. The sections were rinsed thrice with PBS and incubated with anti-AR (ab133273, Abcam), anti-PCNA (GB12010, Servicebio), anti-CD200 (GB114042, Servicebio), and anti-CK15 (GB121648, Servicebio) antibodies at 4 °C overnight. To perform immunohistochemistry, the tissue was covered with horseradish peroxidase (HRP)-labeled goat anti-mouse IgG secondary antibody, followed by incubation for 50 min at room temperature with freshly prepared DAB color development solution (G1212, Servicebio) that was added dropwise. The color development time was controlled under the microscope: the nuclei were re-stained, and the tissue slices were sealed by dehydration to analyze the results under a white light microscope (Olympus, IX73). Immunofluorescence was performed by incubating the tissue with HRP-labeled goat anti-mouse IgG secondary antibody (GB23301, Servicebio) for 50 min at room temperature, followed by the addition of DAPI stain (G1012, Servicebio) to restore the nuclei, then incubated for 10 min at room temperature. Thereafter, an autofluorescence quencher was added. The tissue was then blocked and subjected to fluorescence microscopy (Nikon, Eclipse C1) to capture images.

### Real-time PCR

Total RNA was extracted from back skin tissues using TRIzol reagent (9108, Takara). The cDNA was synthesized using the PrimeScript RT Reagent Kit (RR047A, Takara). To analyze relevant mRNA expression, quantitative PCR was performed on a CFX96 Real-Time PCR Detection System (Bio-Rad) using TB Green Premix Ex Taq (RR820A; Takara). The PCR cycling conditions included initial denaturation at 95 °C for 10 min; followed by 40 cycles of denaturation at 95 °C for 15 s, annealing at 60 °C for 20 s, stretching at 72 °C for 10 s; and a final extension at 72 °C for 5 min. The expression of each mRNA was normalized relative to the expression of GAPDH using the ΔCt method. All reactions were performed in triplicate, and at least three independent experiments were conducted. The primer sequences used in this study are listed in Table 1.

**Table 1:** Primers used for real-time PCR

Primer name	Sequence 5’-3’
*JUND*	Forward: CCATCGACATGGACACGCAA Reverse: CAGCTCGGTGTTCTGGCTTT
*P53*	Forward: CCCCTGTCATCTTTTGTCCCT Reverse: AGCTGGCAGAATAGCTTATTGAG
*Nur77*	Forward: GAGTTCGGCAAGCCTACCAT Reverse: GTGTACCCGTCCATGAAGGTG
*C-FOS*	Forward: GCAGCCATCTTATTCCGTTCC Reverse: CCAGGTCAGCTTCGCAAGG
*HSP27*	Forward: TCTCTATCCCATGATGGCATCC Reverse: CTCAACTCTGGCTATCTCTTCCT
*NFκB*	Forward: ATGGCAGACGATGATCCCTAC Reverse: CGGAATCGAAATCCCCTCTGTT
*CREB*	Forward: AGCAGCTCATGCAACATCATC Reverse: AGTCCTTACAGGAAGACTGAACT
*NFAT-2*	Forward: GGAGAGTCCGAGAATCGAGAT Reverse: TTGCAGCTAGGAAGTACGTCT
*AR*	Forward: TCCAAGACCTATCGAGGAGCG Reverse: GTGGGCTTGAGGAGAACCAT
*GAPDH*	Forward: TGTGTCCGTCGTGGATCTGA Reverse: TTGCTGTTGAAGTCGCAGGAG

### Western blotting

Total protein was extracted using RIPA buffer (AR0102, Boster) supplemented with a protease inhibitor cocktail (HY-K0010, MCE) and a phosphatase inhibitor (AR1183, Boster). The concentration of the extracted proteins was measured using a Bicinchoninic Acid Protein Assay Kit (PC0020, Solarbio), and equal amounts of extracted proteins were loaded onto a sodium dodecyl sulfate-polyacrylamide gel and subject to electrophoresis. The separated proteins were then transferred onto polyvinylidene fluoride membranes (IPVH00010; Millipore) for immunoblotting. The membranes were blocked with 5% BSA blocking buffer (SW3015, Solarbio) and incubated overnight at 4 °C with specific primary antibodies, including anti-AR antibody (ab108341, Abcam), anti-phosphorylated c-Raf (Ser338) (56A6) antibody (9422T, Cell Signaling Technology), anti-c Raf antibody (7422T, Cell Signaling Technology), anti-phosphorylated MEK1/2 (Ser217/221) 41G (9) antibody (9154T, Cell Signaling Technology), anti-MEK1/2 antibody (8727T, Cell Signaling Technology), anti-phosphorylated (Erk1/2) /Thr202/Tyr (204) (197G2) antibody (4695T, Cell Signaling Technology), anti-Erk antibody (4377T, Cell Signaling Technology), anti-Ras antibody (67648T, Cell Signaling Technology), anti-c-Fos antibody (2250T, Cell Signaling Technology), and anti-β-actin antibody (10494-1-AP, Proteintech). After washing, the membranes were incubated with an HRP-coupled secondary antibody (SA00001-2, Proteintech) for 1 h. Protein expression was detected using an ECL western blotting substrate (PE0010, Solarbio), and the membranes were visualized using a membrane imaging system (Clinx, ChemiScope S6).

### Transcriptome sequencing

The total RNA extraction procedure for plant samples was conducted using an RNAprep Pure Plant Kit (Tiangen, China) in strict accordance with the manufacturer’s instructions. Conversely, total animal RNA was extracted using TRIzol Reagent (Life Technologies) according to the manufacturer’s instructions. The concentration and purity of RNA were determined using a NanoDrop 2000 (Thermo Fisher Scientific, Wilmington, DE, USA). RNA integrity was assessed using an RNA Nano 6000 Assay Kit on an Agilent Bioanalyzer 2100 system (Agilent Technologies, USA). RNA samples, comprising 1 μg RNA per sample, were used as input material for the RNA sample preparations. Subsequently, sequencing libraries were generated using the Hieff NGS Ultima Dual-mode mRNA Library Prep Kit for Illumina (Yeasen Biotechnology Co., Ltd., Shanghai, China) following manufacturer’s guidelines. Index codes were assigned to attribute sequences to each sample. Lastly, PCR products were purified using the AMPure XP system, and library quality was assessed using the Agilent Bioanalyzer 2100 system. The libraries were sequenced on an Illumina NovaSeq platform in accordance with the manufacturer’s instructions to generate 150 bp paired-end reads. The raw reads were subjected to further processing through a bioinformatic pipeline tool, the BMKCloud (www. biocloud. net) online platform.

### Statistical analysis

Statistical analyses were performed using GraphPad Prism 8. The experimental data were reported as the mean ± standard deviation (SD) and were analyzed using Student’s t-test. The *P*-values are denoted on graphs as asterisks (^*^) to represent the level of significance (*P* < 0.05; *P* < 0.01; *P* < 0.001).

## Results

### Identifcation of huc-mscs and hUCMSCs-Exos

**Figure 1:**
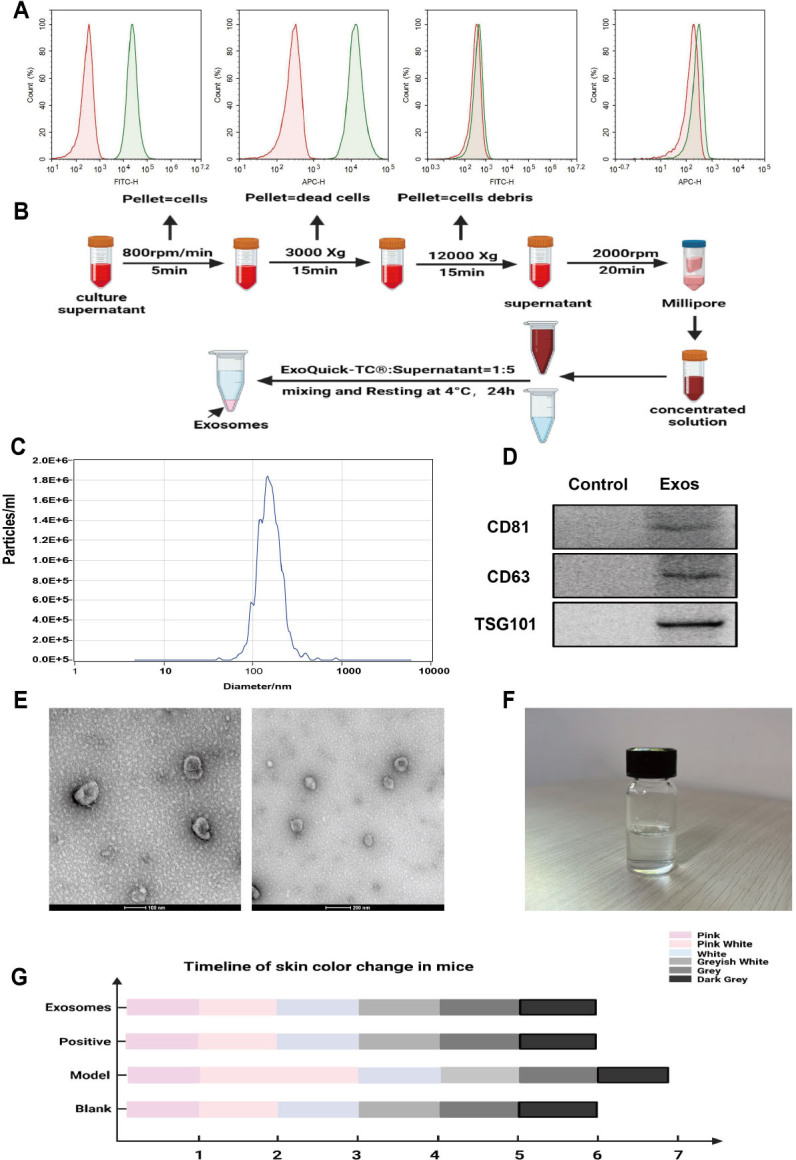
Characterization of hUCMSCs and hUCMSCs-Exos. A. Identifcation of huc-MSCs with CD45, CD73, CD105, and HLA-DR. B. Diagram of exosome extraction. C. NanoSight. D. Western blot. E. HUCMSCs-Exos were identifed by electron microscope, Scale bar = 100 μm. F. Real product photo of Exosome gel. G. Timeline of skin color change in each group. MSC, mesenchymal stem cells.

In the third generation, the cells were homogenized and stabilized before being subjected to the examination of their immunophenotypes using flow cytometry. The analysis revealed that these cells were positive for CD73 and CD105 but negative for CD45 and HLA-DR (Figure 1A). To obtain hUCMSCs-exos, serum-free hUCMSCs medium was processed according to the operating instructions of the ExoQuick-TC® kit after concentrating the supernatant using ultrafiltration tubes (Figure 1B). The diameter size distribution and particle concentration of hUCMSC-Exos were assessed using Nanovision analysis, as depicted in Figure 1C. Western blotting results indicated that hUCMSC-Exos expressed the exosomal surface markers, including CD81, CD63, and TSG101 (Figure 1D), and TEM data further revealed that the hUCMSC-Exos were disc-shaped with a diameter between 30 and 100 nm (Figure 1E). Taken together, these findings suggest that hUCMSC-Exos were successfully isolated in this study.

### Hucmscs-exos-hydrogel complexes at different temperatures

The image of the prepared hUCMSCs-Exos hydrogel is presented in Figure 1F. The appearance of the hydrogel was clear and transparent, and it existed as a colorless transparent liquid that exhibited good fluidity at 4 °C. Notably, the fluidity of the hydrogel was inversely correlated with the temperature, whereby lower temperatures were associated with improved fluidity. Specifically, at 35 °C, the hydrogel reached a semi-solid state and ceased to flow.

### HUCMSCs-Exos promotes hair growth in mice

**Figure 2:**
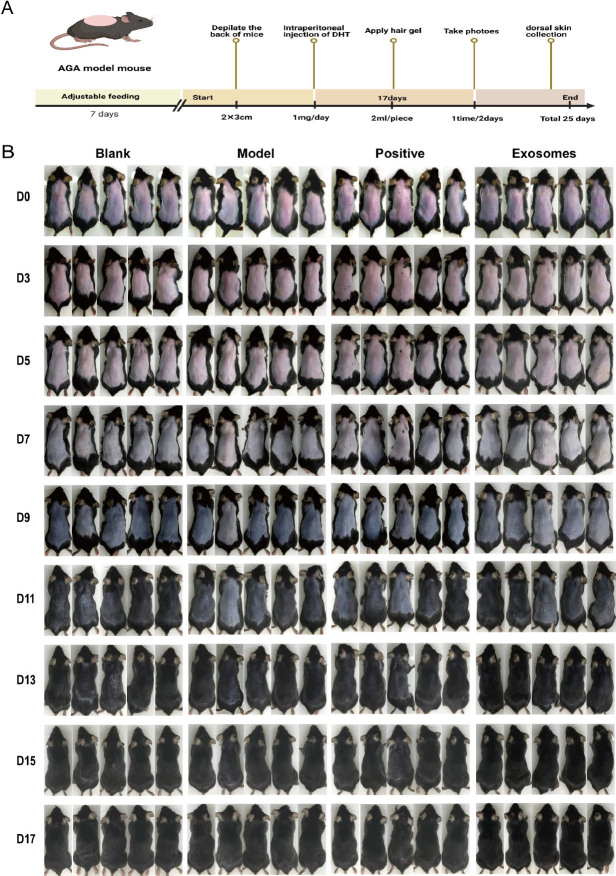
Hair growth records. A. Flow chart of the AGA model constructed with dihydrotestosterone and exosome hydrogel treatment model mice. B. Photographs of hair growth were recorded every 2 days. AGA, Androgenetic alopecia.

**Figure 3:**
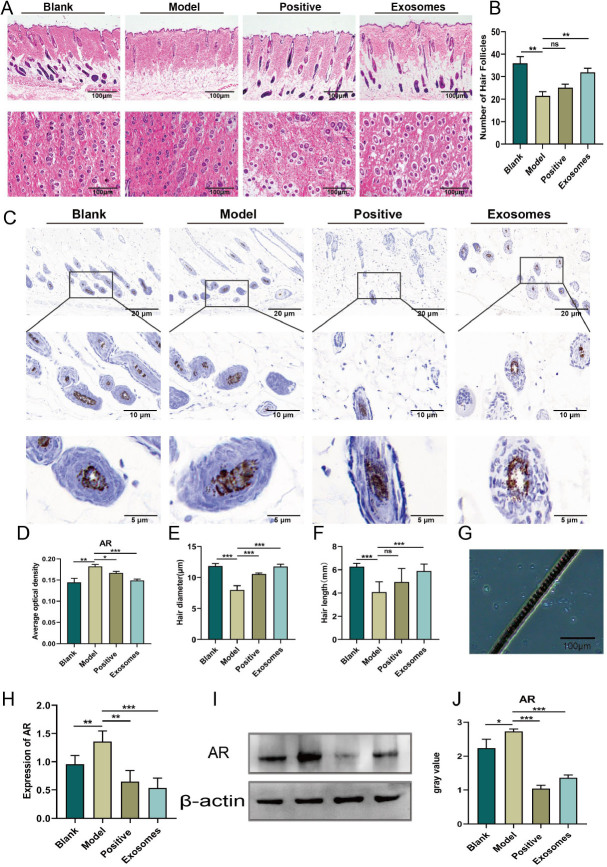
Exosomes reverse dihydrotestosterone-induced AR hyper-expression and promote hair growth. A. HE staining of mouse skin in each group, Scale bar = 100 μm. B. Comparison of hair follicle number in each group. C. Immunohistochemistry of AR expression in hair follicle cells in each group, Scale bar = 20 μm, Scale bar = 10 μm, Scale bar = 5 μm. D. Comparison of average optical density values of AR immunohistochemistry in each group. E. Comparison of new hair diameter in each group. F. Comparison of new hair length in each group of mice. G. Hair diameter detection under microscope. H. Comparison of AR mRNA expression in each group of mice. I. Western blot (WB) analysis of AR abundance in skin tissue of each group of mice. J. Relative AR protein abundance was determined by quantifying band density with ImageJ software. The GAPDH protein expression of each sample was normalized to the ratio to obtain semi-quantitative results. ^*^P < 0.05; ^**^P < 0.01; ^***^P < 0.001.

Initially, we induced a resting phase in the hair cycle of mice by depilating their backs and treated each mouse with DHT at a dose of 1 mg per day intraperitoneally for 17 days to establish an AGA model. The aim was to explore the effect of the hUCMSC-Exos hydrogel on hair growth. The blank group was left untreated, the positive control group was treated twice daily with minoxidil, and the exosome group received twice-daily applications of the hUCMSC-Exos hydrogel (Figure 2A). Hair growth photographs of mice from each group were captured every 2 days and are displayed in Figure 2B. Compared to the blank group, the hair growth of mice in all other groups was inhibited to different degrees, with the model group experiencing the most notable impairment. On day 5 of the experiment, the skin of mice in the model group remained pink compared to that in the blank group, indicating that HFs were still in the resting phase. However, compared to the model group, the skin color of mice in the exosome group turned from pink to gray, and the hair growth entered the growth phase earlier (Figure 1G). After 11 days, hair growth was observed by the naked eye. On day 17, the skin tissue of the mice was excised and stained with hematoxylin-eosin (HE), as shown in Figure 3A. The transverse section of HE showed that, compared to the model group, the blank group displayed more proliferated HFs with a higher count. In contrast, the model group’s HFs were thinner and smaller, and their count was significantly reduced compared to those of the blank group. In comparison, the minoxidil and gel groups exhibited a more considerable number of HFs that were densely distributed, and the HFs proliferated internally. Longitudinal sections showed that the HFs in the blank group were more numerous and morphologically intact. In contrast, the HFs in the model group were shorter and sparser. The lower ends of the HFs were degraded, the hair papillae were thinner, and the inner hair root sheaths were partially lost, resulting in rod-shaped ends. Conversely, both the minoxidil and exosome groups displayed a greater number of HFs, which were longer and larger in size. Statistical analyses showed significant differences in the number of HFs, hair length, and hair diameter between the groups (Figure 3B, 3E, and 3F). Specifically, compared to the blank group, the model group exhibited a decrease in the number of HFs (*P* < 0.01) and an increase in both hair length (*P* < 0.001) and hair diameter (*P* < 0.001). Furthermore, the exosome group exhibited an increase in the number of HFs (*P* < 0.01), hair diameter (*P* < 0.001), and hair length (*P* < 0.001) compared with those in the model group. In contrast, the positive control group exhibited no significant differences in the number of HFs and hair length when compared with those in the model group. However, hair diameter (*P* < 0.001) was found to be significantly higher. Overall, both minoxidil and exosomes were found to be effective in improving HF miniaturization caused by DHT and maintaining normal HF morphology.

### Exosomes reverse dht-induced androgen receptor (ar) overexpression

DHT has been shown to impede hair regeneration *via* AR and to increase AR expression in several organs of male mice, including the brain, hippocampus, prostate and kidney.^[20]^ To determine whether the mice were responsive to androgens, skin was taken at the end of the experiment and immunohistochemically stained with anti-AR antibodies. Expression of AR was observed in the hair follicle tissue of mice in all groups (Figure 3C). The mean optical density values of AR in the skin of model mice treated with DHT were significantly higher than those in the skin of mice in the blank and exosome groups; in addition, the AR optical density values of mice in the positive control group were also reduced compared with those in the model group (Figure 3D). The PCR results confirmed this finding, and the expression of AR in the nuclei of hair papillae of mice in the model group was higher than those in the other two groups. (Figure 3H), indicating that DHT stimulated AR expression and exosomes partially reversed this activation. western blotting results were consistent with the PCR results, and AR expression gray values were significantly higher in the model group mice than in the positive control and exosome groups (Figure 3I, 3J). The results of PCR and western blot analysis indicated that AR was activated in HFs. Overall, these findings map the expression of AR in C57BL/6 mice in response to Hair Follicles and demonstrate that this expression is androgen sensitive.

### Exosomes enhance the stemness of HFSCs and promote the proliferation of hair papilla cells

**Figure 4:**
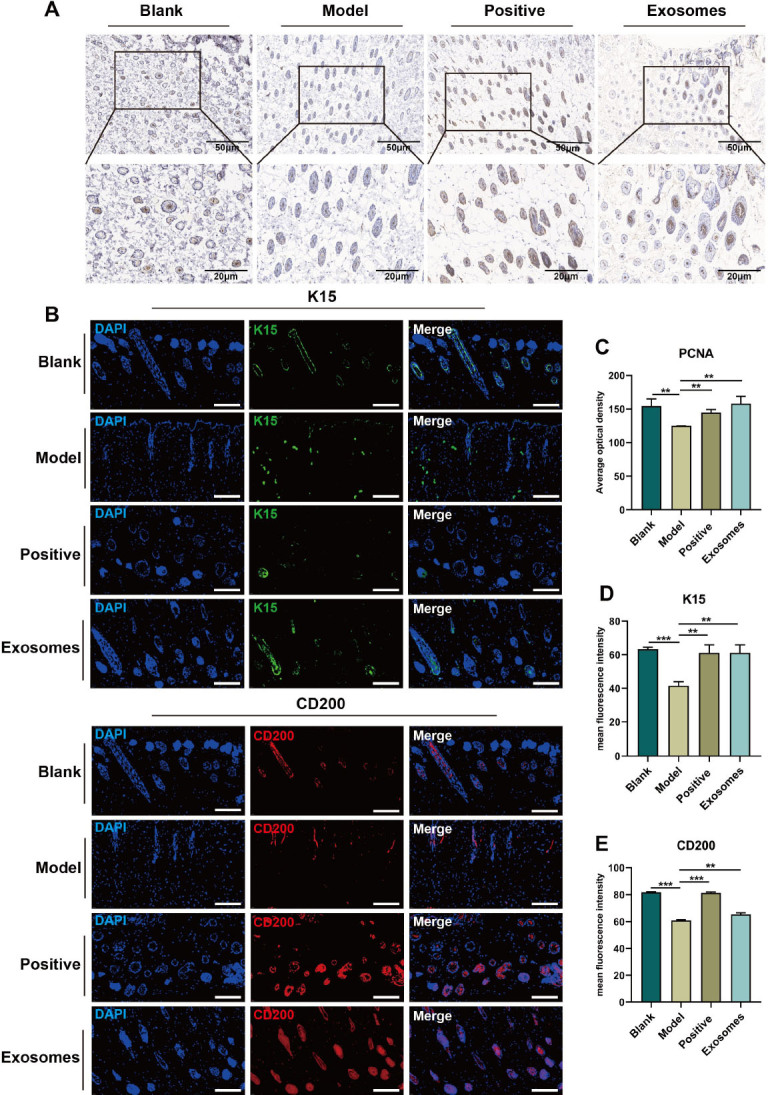
Exosomes enhance the stemness of hair follicle stem cells and promote the proliferation of hair follicle cells. A. Immunohistochemistry of PCNA expression in hair follicle cells of each group, Scale bar = 50 μm, Scale bar = 20 μm. B. Representative immunofluorescence images of K15 (green), CD200 (red) and 4’, 6-diamidino-2-phenylindole (DAPI) expression in hair follicle stem cells, Scale bar = 100 μm. C. Quantification of PCNA expression is represented by average optical density. D. Quantification of CD200 fluorescence intensity by ImageJ software. E. Quantification of K15 fluorescence intensity by ImageJ software. ^**^P < 0.01; ^***^P < 0.001.

Hair papilla cell proliferation was observed using an anti-PCNA antibody through immunohistochemical staining (Figure 4A). Hair papilla cells proliferated to different degrees in the skin tissue of all mice groups, and the protein expression of PCNA was lower in the model, positive control, and exosome groups than in the blank group. The expression of PCNA (*P* < 0.05) was augmented in the positive control and exosome groups compared to that in the model group. Conversely, the expression of PCNA was reduced in the positive control group compared to that in the exosome group, although the difference was not statistically significant (Figure 4C). Previous research suggested that CD200, cytokeratin 15, and CD34 could be utilized as markers of HF epithelial stem cells.^[21]^ CD200 is mainly expressed around the bulge of the HF and in the root lateral ectodermal sheath. Therefore, immunohistochemical staining using the CD200 antibody was employed to assess the expression of HF epithelial stem cells in the bulge region. In this study, CD200 antibody immunohistochemical staining was employed to assess the proliferation and activation of HF epithelial stem cells in the bulges. Immunofluorescence labeling was employed to evaluate the stemness of HFSCs, and HFSC-specific markers, K15 and CD200, were chosen (Figure 4B). The data indicated that the mean fluorescence intensity of CD200 protein expression in HFSCs of mice in the model group was lower than that in the blank group (*P* < 0.001), and CD200 expression was significantly higher in HFSCs of mice in the exosome group than in the HFSCs of mice in the model group (*P* < 0.05) (Figure 4D). Similarly, the mean fluorescence intensity of K15 in HFSCs of mice in the blank group exceeded that in the model group (*P* < 0.001) Furthermore, in the exosome group, the mean fluorescence intensity of K15 protein expression in HFSCs of mice was significantly higher than that of the model group (*P* < 0.05) (Figure 4E). Moreover, minoxidil treatment was observed to enhance the expression of K15 (*P* < 0.05) and CD200 (*P* < 0.001) in HFSCs.

### Exosomes significantly upregulate genes related to the function of transduction mechanisms

**Figure 5:**
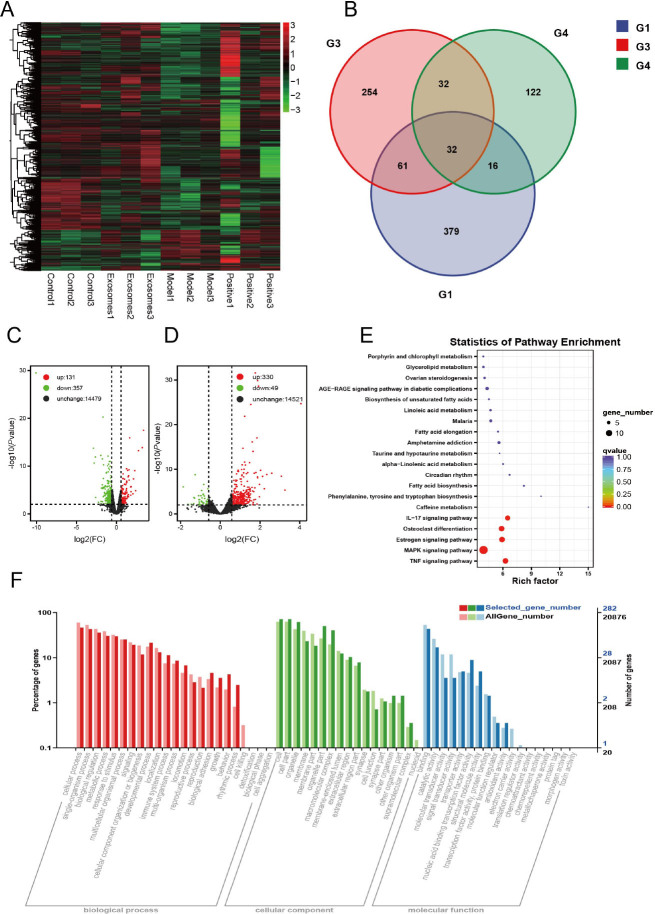
Skin tissue transcriptome sequencing. A. Heat map of skin gene expression clustering in each group of mice. B. Wayne plot of differentially expressed gene set G1: Blank *vs*. Model G3: Model *vs*. Exosomes G4: Model *vs*. Positive. C. Volcano plot, Blank *vs*. Model. D. Volcano plot, Model *vs*. Exosomes. E. Model *vs*. Exosomes upregulated gene KEGG enrichment graph. F. Model *vs*. Exosomes GO classification graph. ^*^P < 0.05; ^**^P < 0.01; ^***^P < 0.001.

Transcriptome sequencing was used to explore the pathway by which hUCMSC-Exos enhanced the expression of stemness-related proteins in HFSCs. The RNA-seq data underwent screening, normalization, and clustering, among other procedures, to construct a heatmap (Figure 5A). Clustering analysis of gene expression levels between different samples indicated a high correlation between groups. Differentially expressed gene (DEG) analysis revealed that in comparison to the model group, 149 genes were significantly upregulated and 87 genes were significantly downregulated in the blank group. Meanwhile, in comparison to the exosome group, 330 genes were significantly upregulated and 49 genes were significantly downregulated in the model group. Table 2 presents the number of DEGs in different groups (P-value = 0.01, FC = 1.5). Functional enrichment of COG and KOG genes for differential genes upregulated in the exosome group compared to those in the model group revealed that these DEGs were mainly involved in signal transduction mechanisms. KEGG Pathway analysis of these differential genes showed that they were mostly enriched in the mitogen-activated protein kinase (MAPK) pathway.

**Table 2:** Statistics on the number of differential gene expression

Group	DEGs-total	DEGs-up	DEGs-down
Control *vs*. Model	488	131	357
Model *vs*. Positive	202	171	31
Model *vs*. Exosomes	379	330	49

### Exosomes regulate hair follicle stem cell stemness via the Erk/MAPK pathway

To investigate the involvement of the MAPK pathway in hair growth, the expression of related genes in mouse skin was assessed using qRT-PCR. The model group showed a significant increase in the expression of genes such as c-FOS (*P* < 0.001), CREB (*P* < 0.01), and P53 (*P* < 0.001) compared to that in the blank group, whereas the exosome group showed a significant decrease in the expression of c-FOS (*P* < 0.001), CREB (*P* < 0.001), and P53 (*P* < 0.01) compared to that in the model group. Conversely, the model group displayed a significant decrease in the expression of genes such as NFAT-2 (*P* < 0.001), Nur77 (*P* < 0.001), JUND (*P* < 0.01), Hsp27 (*P* < 0.01), and AP1 (*P* < 0.01) compared with that in the blank group. The exosome group showed a significant increase in the expression of NFAT-2 (*P* < 0.05), Nur77 (*P* < 0.001), JUND (*P* < 0.001), Hsp27 (*P* < 0.01), and AP1 (*P* < 0.001) compared to that in the model group.

To further investigate the signaling pathway that regulates HFSCs’ stemness, we focused on the RAS/ERK pathway for western blot validation, as indicated by the PCR results of gene expression related to the MAPK pathway. Extracellular regulated protein kinases (ERK), including ERK1 and ERK2, are essential for signal transduction from surface receptors to the nucleus. Phosphorylation-activated ERK1/2 translocate from the cytoplasm to the nucleus and mediates the transcription of ATF, Ap-1, c-fos, and c-Jun, which are involved in various biological responses such as cell proliferation and differentiation, cell morphology maintenance, cytoskeleton construction, apoptosis, and cell carcinogenesis. The subsequent western blotting results confirmed that there were no significant differences in the total amounts of c-Raf, Erk1/2, and Mek1/2 among the blank, model, and exosome groups. Nevertheless, the levels of p-Raf (*P* < 0.05), p-Erk1/2 (*P* < 0.001), and Mek1/2 (*P* < 0.001) were lower in the model group than in the blank group, whereas the exosome group exhibited elevated levels of p-Raf (*P* < 0.01), p-Erk1/2 (*P* < 0.001), and Mek1/2 (*P* < 0.001) compared to those in the model group, indicating an increase in the activation of phosphorylation of these proteins. Similarly, the levels of proteins such as RAS (*P* < 0.001) and c-Fos (*P* < 0.05) in the Erk/MAPK pathway were higher in the exosome group than in the model group.

## Discussion

AGA, a prevalent form of hair loss characterized by progressive follicular miniaturization, is attributed to the presence of androgens in genetically predisposed HFs in androgen-sensitive areas.^[22]^ The pathogenesis of AGA has been found to be primarily related to androgen and AR signaling, with nuclear localization of AR being high in dermal papillae of the balding scalp of AGA patients.^[23]^ If left untreated, the condition progresses gradually.^[24]^ Despite the increasing prevalence of AGA, effective treatment modalities with minimal side effects are still lacking. Therefore, exploring new treatment modalities will lead to innovative developments in hair loss treatment.

**Figure 6:**
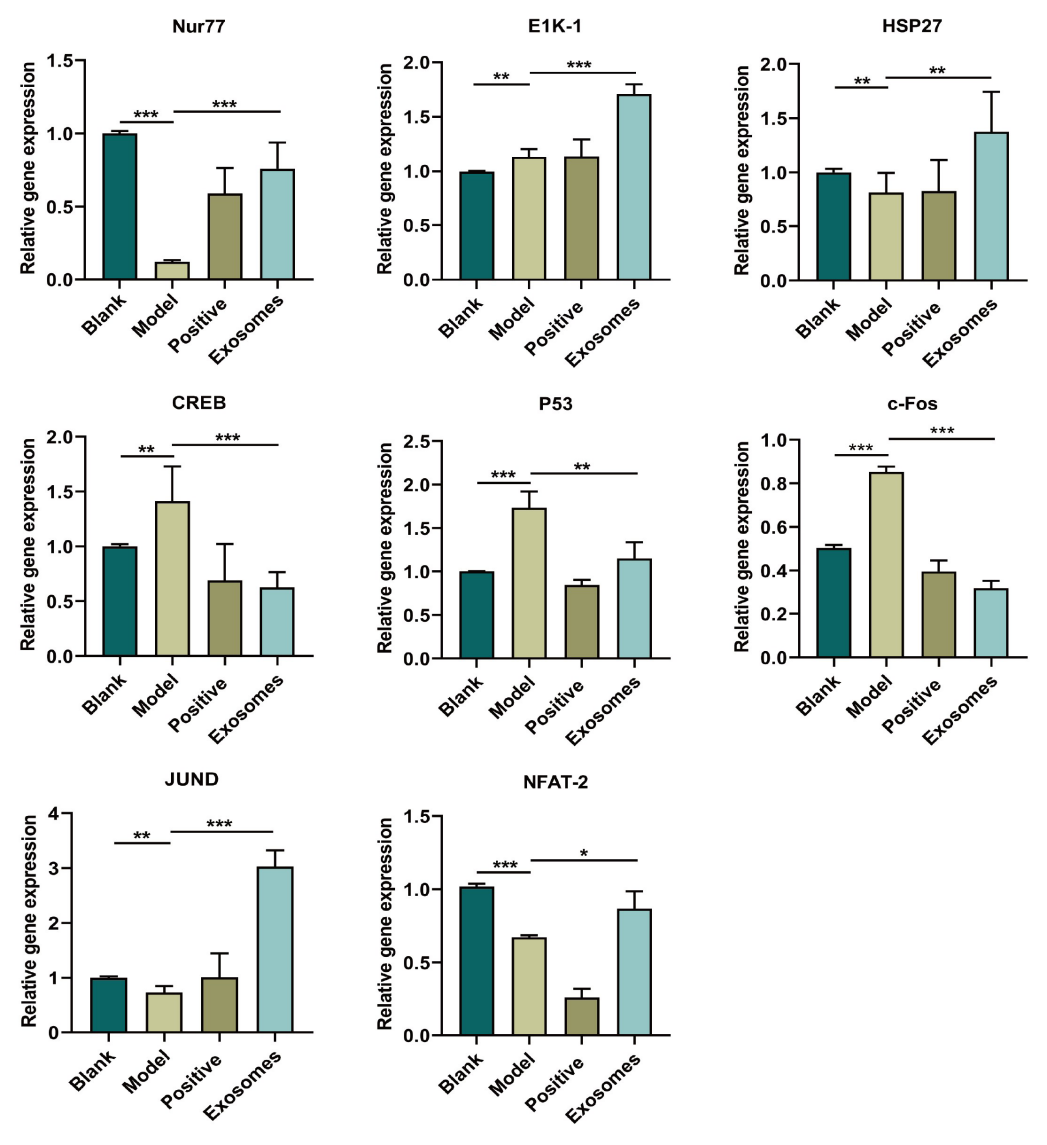
Exosomes promote the expression of ERK/MAPK pathway-related gene mRNA. ^*^P < 0.05; ^**^P < 0.01; ^***^P < 0.001.

**Figure 7:**
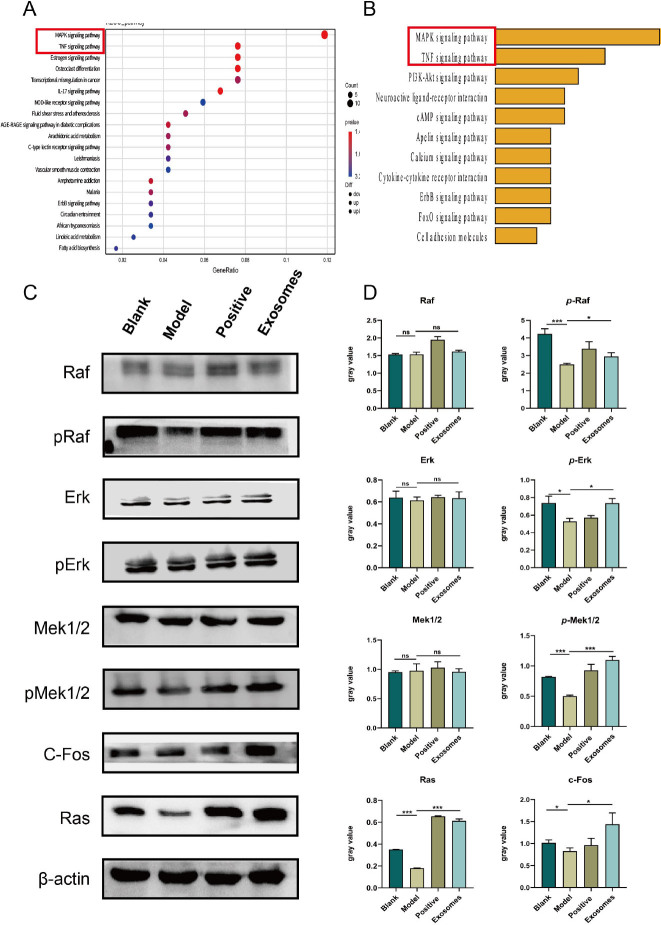
WB validation to increased levels of phosphorylated proteins in the ERK/MAPK pathway. A. Model *vs*. Exosomes upregulated gene KEGG bubble map. B. Model *vs*. Exosomes upregulated gene partial KEGG classification map, Figure S1 in the Supplementary Information shows the upregulated signaling pathway in detail. C. Western blot (WB) assay for RAS/ ERK pathway-associated protein abundance. D. Quantification of band density to determine RAS/ERK pathway-associated protein abundance using ImageJ software. The β-actin protein expression of each sample was normalized to the ratio to obtain semiquantitative results. ^*^P < 0.05; ^***^P < 0.001.

**Figure 8:**
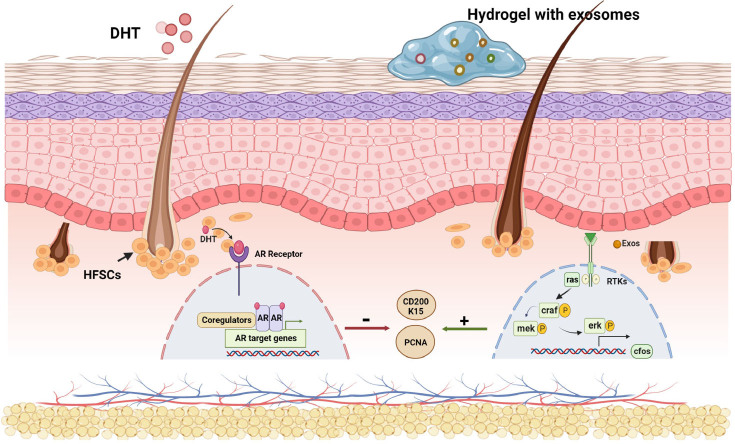
Schematic diagram of the mechanism of exosome treatment of androgenetic alopecia. Dihydrotestosterone miniaturizes hair follicles *via* the androgenic/ androgen receptor pathway, while exosomes regulate hair follicle stem cell stemness *via* the RAS/ERK pathway, thereby promoting hair follicle cell proliferation.

As viable, ready-to-use, cell-free therapies, MSC exosomes have shown promising results in treating various diseases. MSC exosomes can enhance the migration and proliferation of periodontal ligament cells, leading to robust periodontal regeneration through CD73^-^mediated activation of adenosine receptors, promoting pro-survival AKT and ERK signaling.^[25]^ Therefore, to validate the model animals to determine the potential of exosomes to promote hair growth, we developed a mouse model of AGA by administering DHT *via* intraperitoneal injection and combined umbilical cord MSC-derived exosomes with a hydrogel. AR activation in C57BL/6 mice HFs by DHT and AR translocation to the nucleus causes AGA.^[26]^ Immunohistochemistry and western blotting showed increased AR protein expression in the skin tissues of the model group mice. Moreover, they exhibited a lower number of HFs, shorter hair length, and smaller hair diameter than the blank group, indicating successful modeling. However, compared to the model group, exosomes successfully reversed the increase in AR caused by DHT and improved HF miniaturization. Additionally, the expression of PCNA in HFCs of exosome-treated mice was significantly upregulated, indicating that exosomes could promote HF cell proliferation.

A prior investigation reported that individuals with AGA experience follicular stem cell retention in the balding scalp but lack follicular progenitor cells rich in CD200^-^ and CD34^-^ positive cells. This suggests that the cyclical growth of HFs depends on the activation of follicular stem cells.^[21]^ We determined the localization of HFSC stemness *via* immunofluorescence double-labeling and found that the fluorescent signals of K15 and CD200, proteins associated with HFSC stemness, were significantly enhanced in the exosome group compared to those in the model group. Therefore, we hypothesized that exosomes promote hair growth by regulating HFSC stemness.

To further explore the mechanism by which exosomes regulate the stemness of hair follicle stem cells, we took mouse skin tissues for transcriptome sequencing, and the data showed that the differentially expressed genes were mainly enriched in the MAPK pathway. Recent studies indicate that MAPK signaling is involved in regulating stem cell fate.^[26]^ Exosomes have been shown to be endocytosed by human dental pulp stem cells in a dose-dependent and saturated manner through a cavity endocytosis mechanism, triggering the p38 MAPK pathway, which increases the expression of odontogenic differentiation-related genes.^[27]^ In the present study, transcriptome sequencing analysis of mouse skin tissues exhibited that the genes upregulated in the exosome group were mainly enriched in the MAPK/ERK signaling pathway compared to those in the model group. Based on this observation, we propose that exosomes regulate HFSC stemness through the RAS/ERK pathway to promote hair growth in a mouse model of AGA. A schematic diagram illustrating the proposed mechanism is presented below.

In the present study, 5% minoxidil was selected as the positive control. Interestingly, contrary to previous studies, minoxidil was found to reverse the increase in AR levels caused by DHT, suggesting an anti-androgen component.^[28]^ However, there was no difference in the levels of proteins associated with the ERK/MAPK pathway between the positive control and blank groups and the model group, suggesting that minoxidil may not act as an anti-androgen *via* this pathway.

Nonetheless, the present study has certain limitations that should be acknowledged. First, despite the validation of the hair growth-promoting effect of exosomes, the composition of exosomes is intricate, and it remains unclear which specific components are primarily responsible for their therapeutic efficacy. Therefore, it is necessary to further identify the active ingredients in exosomes in future research. Second, the combination of exosomes with hydrogels can significantly decelerate the degradation of protein-like constituents and enhance the bioavailability of exosomes through a slow-release effect, which can potentially augment their therapeutic efficacy. Nevertheless, no separate exosome group was established, and it could not be determined whether the combination of exosomes with hydrogel leads to superior hair growth-promoting effects.

## Supplementary Material

Supplementary Material Details
