# Supplementary figures and images for: Exosomes derived from Umbilical cord mesenchymal stem cell promote hair regrowth in C57BL6 mice through upregulation of the RAS/ERK signaling pathway
Source: J Transl Int Med. 2024 Nov 6;12(5):478–94. doi: 10.1515/jtim-2024-0012 (PMC11538887; doi:10.1515/jtim-2024-0012)

Supplementary Material

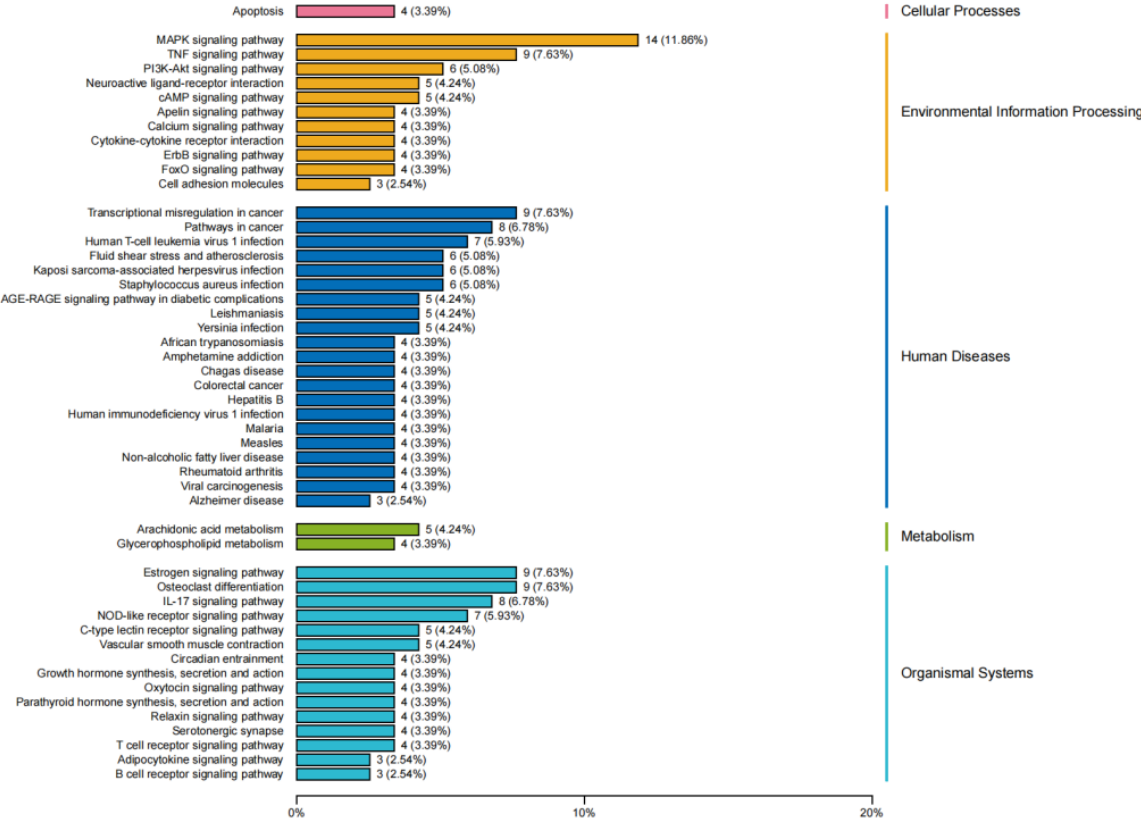

Figure S1: The upregulated signaling pathway.

Supplement: Supplementary file 1 — Supplementary Material Details [file jtim-2024-0012_sm.zip › jtim-2024-0012_sm/3 JTIM-D-23-00129 SI.pdf]
